# Water Deficits Do Not Improve Fruit Quality in *Grapevine Red Blotch Virus*-Infected Grapevines (*Vitis vinifera* L.)

**DOI:** 10.3389/fpls.2020.01292

**Published:** 2020-08-21

**Authors:** Alexander D. Levin, Achala N. KC

**Affiliations:** ^1^Department of Horticulture, Oregon State University, Corvallis, OR, United States; ^2^Southern Oregon Research and Extension Center, Oregon State University, Central Point, OR, United States; ^3^Department of Botany and Plant Pathology, Oregon State University, Corvallis, OR, United States

**Keywords:** deficit irrigation, viral disease, plant water status, gas exchange, grapevine physiology, plant-virus interaction, water deficits, secondary metabolism

## Abstract

Although deficit irrigation is used to improve fruit quality in healthy grapevines, it can potentially amplify negative effects of viral disease and reduce fruit quality in Grapevine Red Blotch Virus (GRBV) infected grapevines. Therefore, a 2-year field experiment was conducted to understand the interaction between GRBV infection and water deficits on disease development and vine physiology. Well-watered (WW) vines were irrigated at 100% of estimated crop evapotranspiration (ET_c_), while water deficit (WD) vines received water at 66 and 50% ET_c_ in 2017 and 2018, respectively. Healthy (GRBV-) and infected (GRBV+) vines were confirmed by PCR assays. There were no significant effects of water deficits on foliar symptom onset in either year, but more severe water deficits in 2018 resulted in a more rapid symptom progression. GRBV+ vines had a higher Ψ_stem_ compared to GRBV- vines, but the effects of virus only appeared post-veraison and corresponded to decreased leaf gas exchange. In general, vine vegetative and reproductive growth were not reduced in GRBV+ vines. Yields were highest in WW/GRBV+ vines due to larger clusters containing larger berries. Consistent treatment effects on berry primary chemistry were limited to sugars, with no interactions between factors. Water deficits were able to somewhat increase berry anthocyanin concentration in GRBV+ fruit, but the effects were dependent on year. By comparison, virus status and water deficits interacted on skin tannins concentration such that they were decreased in WD/GRBV+ vines, but increased in WD/GRBV- vines. Water deficits had no effect on seed phenolics, with only virus status having a significant diminution. Although keeping GRBV+ vines well-watered may mitigate some of the negative effects of GRBD, these results suggest that water deficits will not improve overall fruit quality in GRBV+ vines. Ultimately, the control of fruit ripening imparted by GRBV infection seems to be stronger than abiotic control imparted by water deficits.

## Introduction

Since its discovery in 2008 in California (Calvi, 2011), Grapevine Red Blotch Disease (GRBD)—and its recently confirmed causal agent Grapevine Red Blotch Virus (GRBV) (Yepes et al., 2018)*—*has significantly impacted several major grape-growing areas in the United States (Sudarshana and Zalom, 2017). To date, many research groups have been actively investigating various aspects of GRBD, but most have been focused either on identification and detection (Krenz et al., 2012; Al Rwahnih et al., 2013; Buchs et al., 2018; Romero et al., 2019), genetics and virology (Al Rwahnih et al., 2015; Sudarshana et al., 2015), vector biology and transmission (Bahder et al., 2016; Preto et al., 2018a; Preto et al., 2018b), or disease epidemiology and spread (Cieniewicz et al., 2017; Cieniewicz et al., 2018; Dalton et al., 2019). However, no agronomic studies have been conducted testing interactions between cultural practices and their effects on physiology of GRBV-infected grapevines. Provided with limited information regarding field-level management practices, grape growers have resorted to removing young GRBV-infected vineyards, a time consuming and costly practice (Ricketts et al., 2017). Therefore, more information is necessary regarding the interactive effects of GRBD and cultural practices so that producers can better manage infected vineyards.

GRBV infection has been shown to affect many aspects of vine physiology from leaf metabolism (Wallis and Sudarshana, 2016) to fruit development and ripening (Blanco-Ulate et al., 2017). Ultimately, economic losses are realized through penalties due to a reduction in fruit and wine quality (Ricketts et al., 2017). Early work reported reductions in vegetative growth and canopy development due to what turned out to be GRBV infection (Calvi, 2011). GRBD delays ripening and alters fruit composition through a hypothesized disruption in hormone signaling (Blanco-Ulate et al., 2017). As a consequence, total soluble solids (TSS) are reduced at harvest, and berries of red-fruited has reduced polyphenolic content (Calvi, 2011; Girardello et al., 2019; Martínez-Lüscher et al., 2019).

In many regions affected by GRBD, vineyards are irrigated due to inadequate summer rainfall. Irrigation management is arguably the most effective tool for controlling all aspects of vine growth and development (Williams and Matthews, 1990). While moderate water deficits can advance ripening and improve fruit quality (Matthews and Anderson, 1988; Castellarin et al., 2007; Herrera and Castellarin, 2016), severe water deficits can reduce vegetative growth, photosynthesis, and yield (Williams et al., 2010a; Williams et al., 2010b; Williams, 2012). The onset of ripening in grape involves a transition from xylem to phloem water transport into berries (Greenspan et al., 1994), thus the advancement of ripening due to water deficits may also interact with present GRBV infection given its putative limitation to the phloem (Sudarshana et al., 2015).

Interactions between vine water relations and vascular disease pathogens have been evidenced to varying degrees in previous studies, though the degree of interaction likely depends on pathogen type or tissue localization. Grapevine leaf and petiole hydraulic conductivity were significantly reduced in plants infected with the xylem-limited Pierce’s disease (PD) bacterial pathogen (*Xylella fastidiosa*) compared to healthy plants, and PD susceptibility was shown to increase in water-stressed plants (Choat et al., 2009). A strong interaction between *X. fastidiosa* and water deficit was later observed at the molecular level, whereby pathogen-induced abscisic acid biosynthesis precluded host defense response and increased disease severity (Choi et al., 2013). Nevertheless, disease symptoms may not be tightly correlated to local pathogen presence, as was shown in cases of PD (Gambetta et al., 2007) and recently in grapevines symptomatic of Esca disease (caused by fungal pathogens) (Bortolami et al., 2019). Finally, researchers have recently shown that water deficits interact to a limited degree (or not at all) with viral infection in vines infected with *Grapevine leafroll-associated virus*—a phloem-limited virus like GRBV (El Aou-ouad et al., 2016; El Aou-ouad et al., 2017). Thus, it is likely that there are limited interactions between grapevines infected with GRBV when introduced to water deficits.

Though recent work has begun to characterize GRBD effects on grapevine physiology, these responses have been established by relatively few studies and conducted on a limited number of cultivars. Furthermore, none of the studies have tested GRBV-infected vine responses to any experimental treatments, such as water deficits. Therefore, it is unknown whether water deficits can potentially amplify or hasten the negative effects of GRBD on fruit quality or whether they can attenuate them. Thus, the objective of this study was to subject field grown healthy and GRBV infected grapevines to water deficits over several seasons to test the hypothesis that there is an interactive effect between water deficits and GRBV infection on GRBD symptoms, overall vine growth, and fruit quality.

## Materials and Methods

### Location, Plant Material, and Vineyard Management

The experiment was conducted in a section of a 5-ha commercial *Vitis vinifera* L. cv. Pinot noir vineyard located in the Rogue Valley AVA near Jacksonville, Oregon (43° 31’N, 122° 96’W) planted in 2009. The vines were grafted on Schwarzmann rootstock (*V. riparia* x *V. rupestris*) and rows oriented north-south. Vine and row spacing was 1.83 and 2.74 m, respectively, for a vine density of approximately 2,000 vines per ha.

In 2017, vines were variably pruned with two to four 10-12 bud canes per vine, while in 2018 vines were all uniformly pruned to only two 10-12 bud canes per vine. Foliage was trained with a vertically shoot positioned trellis, with fixed fruiting wires 0.85 and 1 m above the soil surface, and two pairs of foliage catch wires 0.3 and 0.9 m above the fruiting wires. The canopy was hedged when shoots reached 0.3 m above the top of the trellis. All pest and disease management practices were conducted according to the industry standard.

### Experimental Design

The entire vineyard block were previously mapped for GRBD foliar symptoms at the end of 2016. In 2017, six rows with an even distribution of symptomatic vines were selected for the study. Two irrigation treatments, well-watered (WW) and water deficit (WD), and two virus statuses, healthy (GRBV-) and infected (GRBV+), were arranged in a randomized complete block design with a split-plot factorial treatment structure and five replications. The two outer rows were considered border rows and were treated the same as the four inner rows, but data were only collected from the inner rows. The replications (blocks) were repeated down the rows and were 18 vines long. The irrigation treatment main plots were nine vines long and were arranged in each block such that WW and WD treatments were adjacent between blocks and alternated down the rows. A border vine was left between main plots, resulting in plots of 28 vines (seven vines long * four rows wide). The virus status split plots were vines characterized as either GRBV- or GRBV+ within each irrigation treatment main plot. Multiple vines of each virus status were identified in each main plot, thus each individual experimental unit consisted of two to four vines.

### Irrigation Treatments

The irrigation treatments were characterized by varying water application rate based on estimated crop evapotranspiration (ET_c_). Vineyard ET_c_ was estimated using the following equation: ET_c_ = ET_o_ * K_c_, where ET_o_ is reference ET and K_c_ is the crop coefficient. During the experiment, evaluation of vineyard K_c_ over the course of the growing season was calculated using accumulated growing degree-days (GDD) from April 1 and the VSP-specific equation developed by Williams (2014) and adjusted for row spacing. GDD data were downloaded from the Oregon IPM Center website (http://agsci.oregonstate.edu/oipmc). ET_o_, GDD, and precipitation data were obtained from a weather station located at the Southern Oregon Research and Extension Center less than 3 km from the study site (MDFO, AgriMet, United States Bureau of Reclamation). GDD were calculated using the single sine method with a lower threshold of 10°C.

The irrigation treatments were imposed by varying the number of emitters per vine in the drip line, and the irrigation pump was scheduled to run based on the application rate of the WW treatment plots. Thus, by increasing emitter density in the WW plots, WD plots necessarily received water at a fraction of WW plots. In 2017, WW plots were irrigated with three 4 L/h emitters per vine to replace 100% of estimated ET_c_, while WD plots were irrigated with two 4 L/h emitters per vine, and subsequently received water at 66% of estimated ET_c_. To increase the severity of the water deficits in WD vines relative to WW vines in 2018, WW plots again received water at 100% of estimated ET_c_, but with four 4 L/h emitters per vine, while WD plots continued to be irrigated with two 4 L/h emitters per vine, thus subsequently received water at 50% of estimated ET_c_.

### Identifying Infected Vines

Within each irrigation treatment, healthy and infected vines were identified based on symptoms data in 2016. The identified vines were confirmed for virus status – infected (GRBV+) and healthy (GRBV-) by PCR-based assays. Four petioles from individual vines were collected around veraison in 2017 and total genomic DNA was extracted from combined sample of four petioles per vine. The primer pairs targeting a coat protein (*CP*) gene and a replicase-associated protein (*Rep*) gene fragments (Krenz et al., 2014) were used to amplify in multiplex PCR reactions. The PCR was carried out as suggested for primer pairs *CPfor* and *CPrev*; *Repfor* and *Reprev* (Krenz et al., 2014) in a C1000 Touch Thermal Cycler (Bio-Rad Laboratories, Hercules, CA). A positive and negative DNA samples from previously confirmed vines were used as controls. The resulted amplicons were visualized by electrophoresis on 1% agarose gel stained with GelRED (Biotium, Fremont, CA). The detection of amplicons on agarose gel electrophoresis were considered as virus infected sample (Al Rwahnih et al., 2013; Krenz et al., 2014). The healthy vines were again tested for confirmation at the end of each growing season in 2017 and 2018; and beginning of the season in 2018. At least four diseased and healthy vines were included per treatment per replication except for block 1 treatments as not enough diseased vines were detected in the block.

### Quantification of GRBD Progression and Severity

The progression and severity of GRBD symptom expression in response to the irrigation treatments was quantified in infected and healthy vines using Horsfall-Barratt scale (Horsfall and Barratt, 1945). The scale was used to quantify disease severity as the proportion of symptomatic tissues per vine. The mid-point percentage of the Horsfall-Barratt rating was used for data analysis (Horsfall and Barratt, 1945). Data on disease severity was collected every week after the appearance of first symptom until harvest in both years. The area under disease progress curve (AUDPC) was calculated using the formula: Σ([(x_i_+x_i-1_)/2](t_i_-t_i-1_)), where x_i_ is the mid-point percentage of the Horsfall-Barratt rating at each evaluation time and (t_i_-t_i-1_) is the time between evaluations.

### Vine Water Status Measurements

To quantify responses of vine water status to treatments over time in 2017 and 2018, measurements of midday stem water potential (Ψ_stem_) were made at regular intervals throughout the season. Ψ_stem_ was measured with a pressure chamber (Model 600, PMS Instruments, Corvallis, OR) using a modified method (Levin, 2019). Specifically, Ψ_stem_ measurements were taken between 1300 and 1500 h Pacific Daylight Time (PDT). Leaves chosen at the time of measurement were the most recently expanded, mature leaves, and exposed to direct solar radiation. Leaf blades were covered with an opaque aluminum foil bag, quickly sealed, and allowed to equilibrate for at least 10 min. Once leaves were excised from the plant, time between leaf excision and pressurization was within 30 s. One leaf was measured per plot and used for data analysis.

### Leaf Gas Exchange Measurements

Survey measurements of leaf-level gas exchange were taken with a portable photosynthesis system (LI-6400XT, LI-COR Biosciences, Lincoln, NE) on one leaf per plot across experimental treatments over the course of the growing season from May to September in 2018. Data were collected between 1200 and 1500 h PDT on light-adapted leaves similar to those used for the determination of Ψ_stem_. Irradiance, relative humidity, and temperature were set to match ambient conditions, flow rate was set at 500 µmol/s, and CO_2_ concentration was controlled on the reference chamber at 400 µmol/mol. Analyzers were matched every 20 min.

### Measurements of Vine Growth, Crop Yield, and Quality

Data vines were harvested the day before commercial harvest in each year to evaluate possible interaction effects of GRBD and water deficits on crop yield and quality. Commercial harvest was generally decided when fruit TSS reached approximately 24° Brix. However, whereas in 2017 fruit was contracted for red wine production, in 2018, it was contracted for rosé production. Thus, fruit was harvested two weeks earlier (7 Sept) compared to 2017 (21 Sept).

On the morning of experimental harvest, 20 berries per data vine were sampled from the morning side of the canopy and brought back to the laboratory for analyses described below. Next, all clusters were harvested from vines, counted, and weighed in the field using a calibrated balance (Defender 3000, OHAUS Corporation, Parsippany, NJ). In the laboratory, berries from all vines in each plot were combined in one bag (60–80 per plot) and mixed in the bag by hand, and then 20 berries were subsampled for phenolics analysis.

Remaining berries were crushed in their sample bags, juice decanted and centrifuged. TSS was measured using standard benchtop refractometry. Titratable acidity (TA) and pH were determined using an auto-titrator (Model T50, Mettler Toledo, Columbus, OH). Skin and seed phenolics were determined by Harbertson-Adams assay as described in Casassa et al. (2015) with modifications of Harbertson et al. (2015) and scaled down for a microplate reader as in Heredia et al. (2006). Berries per cluster and cluster fresh weight (FW) were estimated from measured yield components. Shoot number and pruning mass per vine were determined during dormancy.

### Statistical Analyses

The AUDPC values were subjected to analysis of variance using GLIMMIX procedure in SAS (ver. 9.4, SAS Institute, Cary, NC, USA). All other statistical analyses and graphics were conducted in R statistical software (v. 3.6.1; www.R-project.org). Season-long measurements of vine water status and gas exchange were analyzed separately for each year *via* three-way ANOVA for a split-split-plot design using the *lmerTest* package (v. 3.1; Kuznetsova et al., 2017). Main plots were irrigation treatments, split plots were virus status, and split-split plots were measurement dates. The same vines were measured on each date in within a given year. Measurements of vine vegetative growth, yield and yield components, and fruit quality variables were analyzed *via* three-way ANOVA for a split-split-plot design using the same package. Main plots were irrigation treatments, split plots were virus status, and split-split plots were years. The same vines were measured in each year. All means and comparisons among them were conducted using the *emmeans* package using the Tukey multiplicity adjustment method (v. 1.4.3; Lenth, 2019). Graphics were made using *ggplot2* (v. 3.2.1; Wickham, 2016).

## Results

### Environmental Conditions, Irrigation Treatments, and Vine Phenology

Both growing seasons were similar to one another in terms of heat accumulation and evaporative demand but differed widely in precipitation (**Table 1**). In 2017, the 30-year average for accumulated water year precipitation (505 mm) was attained by mid-January (109 days into the water year). In contrast, the pattern of accumulated precipitation departed from the historical average 2 months into the water year (beginning of December), and never recovered in 2018. By budbreak in 2018 (23 April), only 265 mm of precipitation had fallen. Vine phenology was similar between growing seasons, with differences primarily in early season events. Treatments were imposed during the first week of July in both seasons, corresponding to approximately 21 and 34 days after anthesis.

**Table 1:** Evaporative demand and water supply for the 2017 and 2018 seasons.

Year	GDD	ET_o_				Applied water	
			Precipitation			Irrigation treatment	
			10/1^a^ to 3/31	4/1 to 9/30	Total	WW	WD
		*---------------- mm --------------*					
2017	1770 (*105*)	894 (*104*)	717 (*183*)	89 (*78*)	806 (*160*)	126	89
2018	1708 (*102*)	865 (*100*)	241 (*62*)	81 (*71*)	322 (*64*)	188	94

Demand is indicated by accumulated growing degree-days (GDD; base 10C from 1 April to 31 October) and reference ET (ET_o_). Supply is indicated by precipitation and applied water for the entire season and for well-watered (WW) versus water deficit (WD) irrigation treatments. Numbers in parentheses indicate values in percent relative to the 30-year average. ^a^ From previous year.

**Figure 1:**
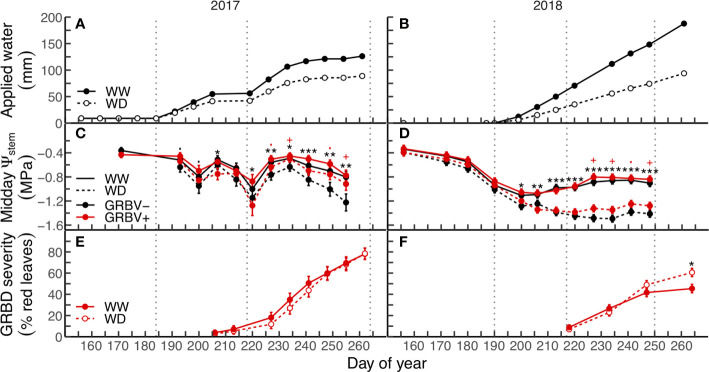
Applied water amounts **(A, B)**, midday stem water potential **(C, D)**, and disease severity **(E, F)** in response to irrigation treatments and virus status (in 2017 and 2018, respectively). Data are means ± 1 standard error (n = 5). Black symbols ‘.’, ‘*’, ‘**’, and ‘***’ represent statistical significance at *p* < 0.10, 0.05, 0.01, and 0.001, respectively, for well-watered (WW) versus water deficit (WD), and likewise, red symbols ‘.’ and ‘+’ are respective to those *p-*values for GRBV- versus GRBV+. Vertical dotted lines represent dates of treatment imposition, veraison, and harvest, from left to right, respectively.

Applied water amounts and their patterns of application varied between years (**Figures 1A, B**). In both seasons, the irrigation treatments were imposed the first week of July, though in 2017, a small amount of water was equally applied to all vines prior to anthesis (**Figure 1A**). Irrigation was cut off for two periods in 2017: one preceding the onset of veraison and the other several weeks before harvest (**Figure 1A**). In contrast, irrigation was applied regularly to vines each week until just prior to harvest in 2018 (**Figure 1B**).

### Responses of Vine Water Status, Disease Progression and Severity

Irrigation treatment and virus status significantly and independently affected vine water status (Ψ_stem_) in each year (**Figures 1C, D**). The deficit irrigation treatment consistently reduced Ψ_stem_ in both years across virus status, with significant reductions in Ψ_stem_ at nearly every sample date following treatment imposition in mid-July. In contrast, the significant effect of virus status on Ψ_stem_ only appeared after veraison in both years regardless of irrigation treatment. While there were no differences in Ψ_stem_ between vines of different virus status preveraison, GRBV+ vines exhibited higher postveraison Ψ_stem_ values by 0.13 and 0.10 MPa in 2017 and 2018, respectively, when averaged across irrigation treatments.

The symptomatic vines from 2016 vineyard mapping were accurately correlated to the PCR-based assay. The vineyard was mapped based on GRBD symptoms for two consecutive years (2015 and 2016), and 95% of the symptomatic vines tested positive for virus. Similarly, 96% of the asymptomatic vines tested negative. The remaining 4% of the vines that were marked asymptomatic in previous years exhibited symptom in 2017 and subsequently tested positive for the virus.

GRBD foliar symptom onset was not affected by irrigation treatments in either year, though first symptoms were observed nearly two weeks preveraison in 2017, compared to just at veraison in 2018 (**Figures 1E, F**). In 2017, no significant interaction between irrigation treatment and virus status was observed for disease progress over time. In contrast, the rate of disease progress on infected vines within the WD treatment was significantly higher compared to the WW treatment in 2018 (*p =* 0.0053).

### Response of Leaf Gas Exchange

**Figure 2:**
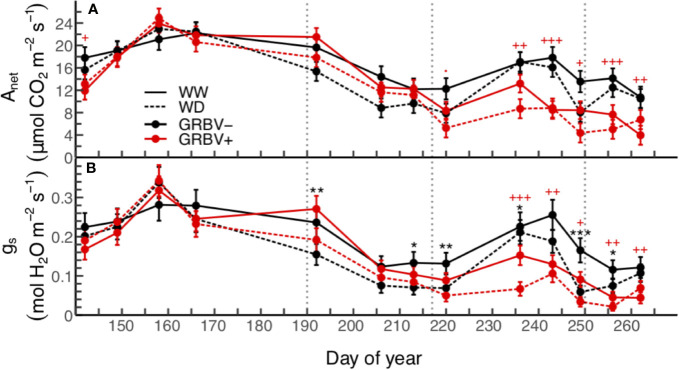
Season-long responses of net carbon assimilation **(A)** and stomatal conductance **(B)** to irrigation treatments and virus status in 2018. Data are means ± 1 standard error (n = 5). Symbols ‘.’, ‘*,’ ‘**’, and ‘***’ represent statistical significance at *p* < 0.10, 0.05, 0.01, and 0.001, respectively, for well-watered (WW) versus water deficit (WD), and likewise, symbols '.', '+', '++', and '+++' are respective to those *p-*values for GRBV- versus GRBV+. Vertical dotted lines in represent dates of treatment imposition, veraison, and harvest, from left to right, respectively.

In general, highest values for leaf gas exchange were measured early/mid-season during the month of June (**Figure 2**). This corresponded to the period from just prior to anthesis through to bunch closure and was prior to irrigation treatment imposition. Net carbon assimilation (A_net_) values ranged from 15 to 23 µmol CO_2_/m^2^/s and stomatal conductance (g_s_) values ranged from 0.195 to 0.320 mol H_2_O/m^2^/s during this time when averaged across all treatments.

Significant differences in A_net_ and g_s_ were observed between irrigation treatments immediately following treatment imposition on 9 July through to harvest (**Figure 2**). Irrigation treatment differences were generally more pronounced in g_s_ compared to A_net_, with 29 to 46% reductions in g_s_ compared to 15 to 36% reductions in A_net_ during this time when averaged across virus status. Overall values for both parameters decreased during this period in part due to an increase in local wildfire activity and subsequent reduction of PAR below saturating levels (data not shown).

Significant differences in both A_net_ and g_s_ were observed as a function of virus status immediately following veraison through post-harvest (**Figure 2**). Notably, reductions in A_net_ due to virus status were observed prior to reductions in g_s_. There was a transient increase in both gas exchange variables across all treatments, due to clearing up of wildfire smoke, but then values dropped toward the end of the season as leaves aged. A_net_ was reduced by 33 to 52%, and g_s_ was similarly reduced by 31 to 66% during this time. In addition, irrigation treatment differences continued into this period, though as before were more pronounced for g_s_ compared to A_net_. Finally, there were no statistically significant interactions between irrigation treatment and virus status for A_net_ or g_s_w when averaged over the course of the whole season or for individual sample dates (*p* > 0.05).

### Response of Vine Vegetative and Reproductive Growth

The change in commercial pruning and canopy management practices from 2017 to 2018 resulted in a highly significant year effect on shoots per vine and shoot FW, but not on pruning mass (**Table 2**). There was a significant irrigation by year interaction effect (*p* = 0.015) on pruning mass whereby there was a larger difference in pruning mass between irrigation treatments in 2018 compared to 2017 (data not shown). There were no other significant main effects of irrigation treatment or virus status (nor interactions) on vegetative growth variables. Nevertheless, to account somewhat for differences in canopy architecture between years, shoots per vine was included in all subsequent analyses of crop yield and quality variables as a co-variable.

**Table 2:** Year effect on vegetative and reproductive growth variables averaged across irrigation treatment and virus status.

Variable	Year		*p*-value
	2017	2018	
*Vegetative*			
Shoots per vine	22.9 ± 0.9	16.0 ± 0.9	<0.001
Shoot FW (g)	49.4 ± 5.2	72.9 ± 6.3	<0.001
Pruning mass (kg/vine)	1.18 ± 0.14	1.20 ± 0.14	0.545
*Reproductive*			
Yield (kg/vine)	4.1 ± 0.5	7.5 ± 0.5	<0.001
Clusters per vine	25.8 ± 1.2	45.3 ± 2.1	<0.001
Clusters per shoot	1.5 ± 0.1	2.3 ± 0.1	<0.001
Cluster FW (g)	141 ± 8	179 ± 8	0.003

Data are means ± 1 standard error (n = 5).

**Figure 3:**
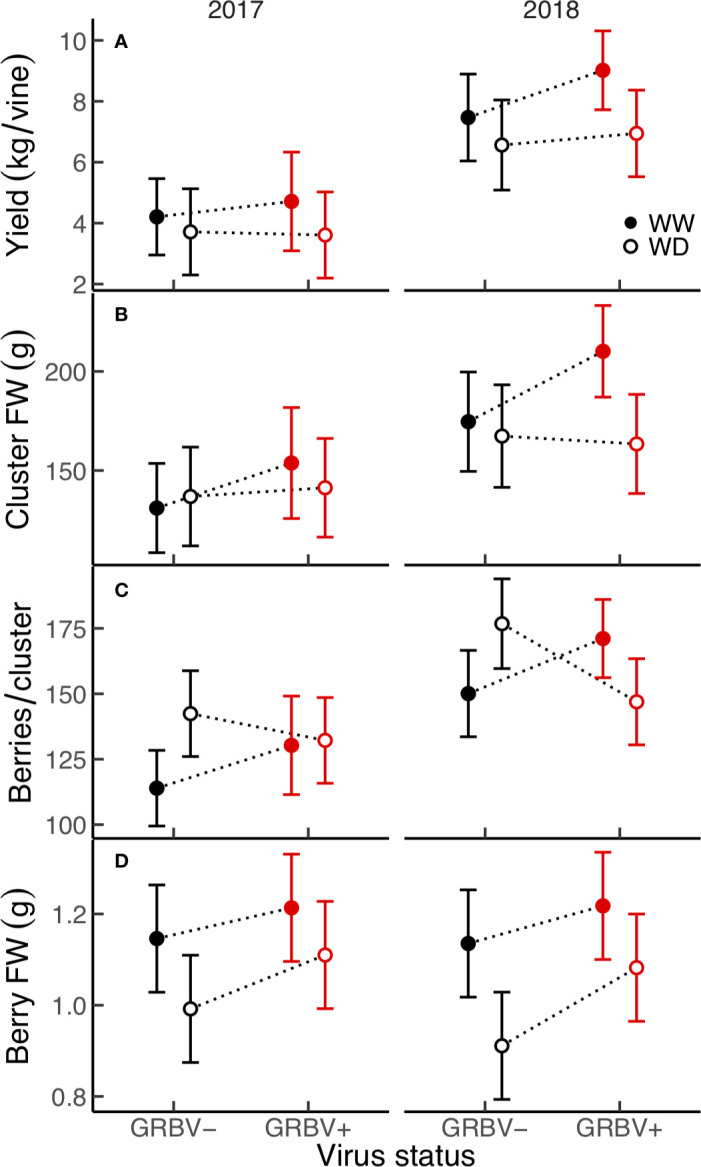
**(A–D)** Treatment interaction plots of yield and select yield components in each year of the study. Data are means with 95% confidence intervals (n = 5). WW and WD are well-watered and water deficit irrigation treatments, respectively.

There were no significant treatment effects on vine yield, clusters per vine, clusters per shoot, and cluster FW, only differences between years (**Table 2**). Yield was a significant function of clusters per vine (R^2^ = 0.78, *p* < 0.001) and of cluster FW (R^2^ = 0.74, *p* < 0.001), all of which were significantly higher in 2018. However, yield and cluster FW trended higher in WW/GRBV+ vines, particularly in 2018 (**Figures 3A, B**).

Independent of year, there was a significant irrigation by virus interaction effect between irrigation treatment and virus status on berries per cluster (*p* = 0.018), though berries per cluster was significantly higher in 2018 compared to 2017 (**Figure 3C**). Averaged across years, there were 21% more berries per cluster in WD/GRBV- vines compared to WW/GRBV- vines, but 7% fewer berries per cluster in WD/GRBV+ vines compared to WW/GRBV+ vines (**Table 4**). Also independent of year, berry FW was decreased in WD compared to WW vines and increased in GRBV+ compared to GRBV- vines (**Figure 3D**). Berries were 14% smaller in WD compared to WW vines when averaged across years and virus status (**Table 3**). Berries were 10% larger in GRBV+ compared to GRBV- vines when averaged across years and virus status, but the effect was not statistically significant (*p* = 0.097).

**Table 3:** Responses of berries per cluster and berry FW to irrigation treatment and given virus status and averaged across virus status.

		Virus		
Variable	Irrigation	GRBV-	GRBV+	Irrigation mean
Berries per cluster	WW	132 ± 5	151 ± 5	141 ± 4
	WD	160 ± 5	140 ± 5	150 ± 4
	*p*-value	0.019	0.460	–^a^
Berry FW (g)	WW	1.14 ± 0.05	1.22 ± 0.05	1.18 ± 0.04
	WD	0.95 ± 0.05	1.10 ± 0.05	1.02 ± 0.04
	*p*-value	–^b^	–^b^	0.043

Data are means (averaged across years) ± 1 standard error (n = 5). WW and WD are well-watered and water deficit irrigation treatments, respectively.

a p-value not computed due to involvement in interaction.

b p-value not computed due to non-significant interaction effect.

**Table 4:** Main effects of irrigation treatment and virus status for primary berry chemistry variables averaged across years.

Treatment	Level	TSS (Brix)	pH	TA (g/L)
Irrigation	WW	23.0 ± 0.2	3.38 ± 0.03	4.07 ± 0.13
	WD	22.0 ± 0.2	3.37 ± 0.03	3.90 ± 0.13
	*p*-value	0.025	0.519	0.263
Virus	GRBV-	23.3 ± 0.2	3.34 ± 0.03	4.11 ± 0.13
	GRBV+	21.7 ± 0.2	3.41 ± 0.03	3.87 ± 0.13
	*p*-value	0.005	0.195	0.126

Data are means ± 1 standard error (n = 5). WW and WD are well-watered and water deficit irrigation treatments, respectively.

### Berry Primary Chemistry

**Figure 4:**
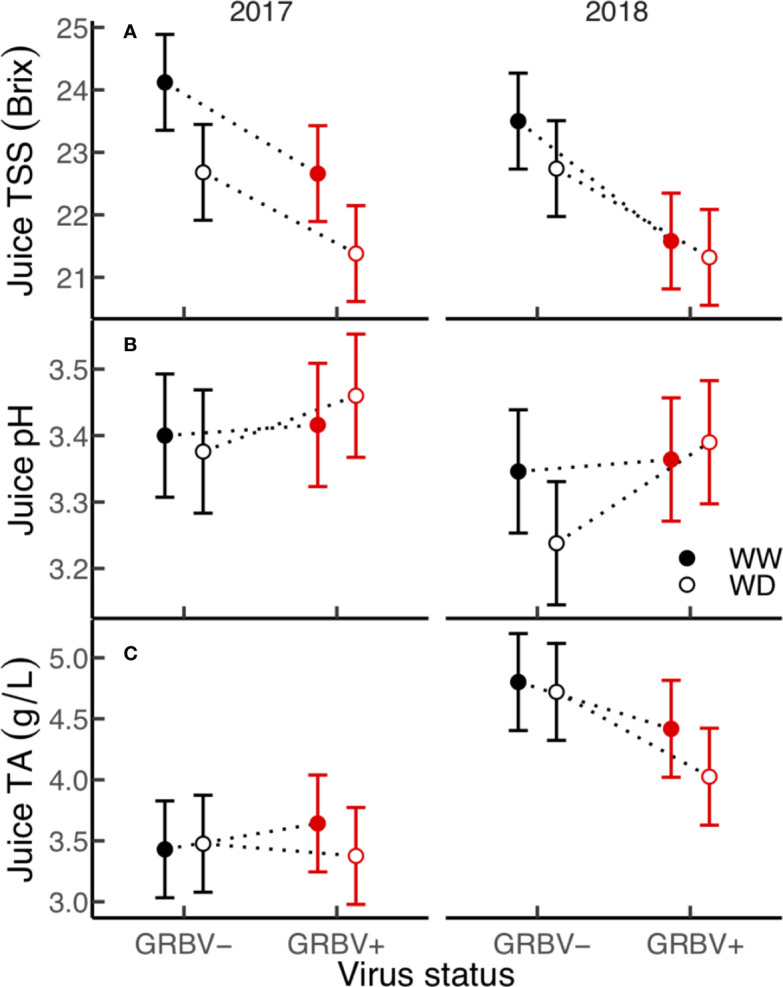
**(A–C)** Treatment interaction plots of berry chemistry variables in each year of the study. Data are means with 95% confidence intervals (n = 5). WW and WD are well-watered and water deficit irrigation treatments, respectively.

Irrigation treatment and virus status consistently and independently impacted berry TSS across both years, but had little effect on pH and TA (**Figure 4**). The effect of virus on TSS was stronger than that of irrigation, with TSS significantly reduced by 1.5 Brix in GRBV+ vines compared to GRBV- vines when averaged across years and irrigation treatments (**Table 4**). By comparison, the WD treatment significantly reduced TSS by 0.9 Brix compared to WW when averaged across years and virus status. Berry pH and TA were not significantly affected by either treatment within a given year nor in a consistent direction across years, and only varied between years.

### Berry Secondary Metabolites

**Figure 5:**
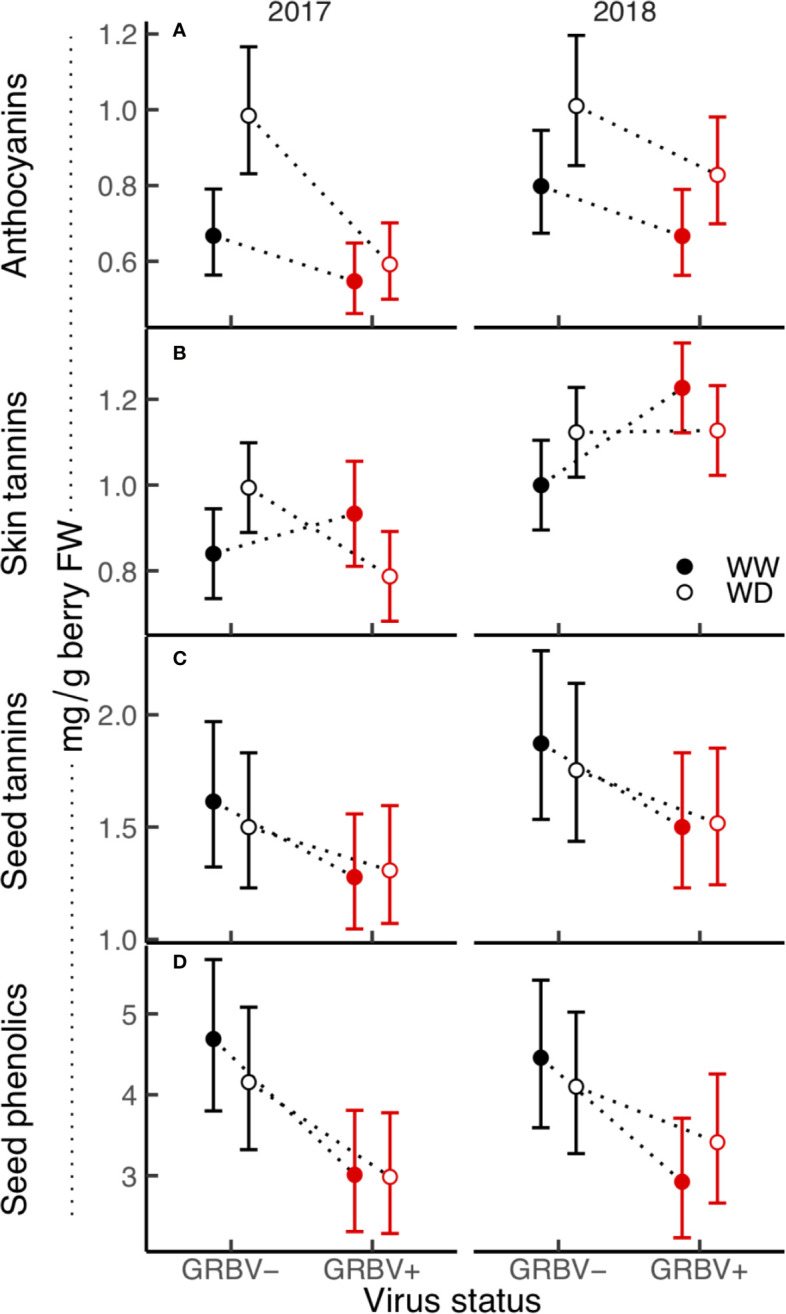
**(A–D)** Treatment interaction plots of berry skin and seed phenolics in each year of the study. Data are means with 95% confidence intervals (n = 5). WW and WD are well-watered and water deficit irrigation treatments, respectively.

In general, the effect of virus status predominated the significant responses in both skins and seeds, though responses of skin secondary metabolites were more variable between years compared to responses of seed secondary metabolites, which were more consistent between years (**Figure 5**). Moreover, there were strong main effects and some significant interactions among experimental treatments for secondary metabolites in berry skins, but relatively few for those found in seeds.

#### Skins: Anthocyanins and Tannins

Berry anthocyanin concentration ranged from 0.55 to 1.01 mg/g berry FW across treatments and years (**Figure 5A**). Notably, there was some interaction between irrigation treatment and virus status in 2017 relative to 2018, resulting in a nearly significant three-way interaction in the ANOVA (*p* = 0.069). Anthocyanin concentration was generally reduced in GRBV+ vines, though the effects of virus status were more pronounced in 2017 compared to 2018, resulting in a significant virus by year interaction in the ANOVA (*p* = 0.044). In GRBV+ vines, anthocyanin concentration was reduced by 30 and 18% in 2017 and 2018, respectively, when averaged across irrigation treatments (**Table 5**). When expressed on a per berry basis (i.e. anthocyanin content), results were similar—reductions of 24 and 7% in 2017 and 2018, respectively, for GRBV+ vines compared to GRBV- vines (data not shown). There was also a marginally significant main effect of irrigation treatment on anthocyanin concentration (*p* = 0.047) that was not present on anthocyanin content (*p* = 0.107). Averaged across virus status and years, anthocyanin concentration was increased by 27% in WD compared to WW vines (**Table 6**), while content was only increased by 9% (data not shown).

**Table 5:** Response of berry anthocyanin concentration to irrigation treatment and virus status in each year individually, and across years.

Treatment	Level	Year		Across-year Mean
		2017	2018	
		*————— mg/g berry FW —————*		
Irrigation	WW	0.60 ± 0.04	0.73 ± 0.05	0.66 ± 0.04
	WD	0.76 ± 0.05	0.91 ± 0.06	0.84 ± 0.05
	*p*-value	–^a^	–^a^	0.024
Virus	GRBV-	0.81 ± 0.05	0.90 ± 0.05	0.85 ± 0.05
	GRBV+	0.57 ± 0.03	0.74 ± 0.04	0.65 ± 0.04
	*p*-value	0.002	0.061	–^b^

Data are means ± 1 standard error (n = 5). WW and WD are well-watered and water deficit irrigation treatments, respectively.

a p-value not computed due to non-significant interaction effect.

b p-value not computed due to involvement in interaction.

**Table 6:** Interaction effect of irrigation treatment and virus status on skin tannins averaged across years.

	Virus	
Irrigation	GRBV-	GRBV+
	*——— mg/g berry FW ———*	
WW	0.92 ± 0.04	1.08 ± 0.04
WD	1.06 ± 0.04	0.96 ± 0.04
*p*-value	0.033	0.057

Data are means ± 1 standard error (n = 5). WW and WD are well-watered and water deficit irrigation treatments, respectively.

Berry skin tannin concentration ranged from 0.79 to 1.23 mg/g FW across treatments and years (**Figure 5B**). The ANOVA resulted in a significant virus by irrigation treatment interaction on tannin concentration (*p* = 0.022), whereby skin tannin was significantly increased by 15% in WD/GRBV- vines compared to WW/GRBV- vines when averaged across years (**Table 6**). Conversely, it was decreased by 11% in WD/GRBV+ vines compared to WW/GRBV+ vines when averaged across years. There was also a significant virus by year interaction (*p* = 0.029), whereby skin tannin concentration was significantly increased in GRBV+ vines in 2018, but not in 2017. When expressed on a per berry basis, skin tannin content scaled with berry size (data not shown).

#### Seeds: Tannins and Total Phenolics

Berry seed tannin concentration ranged from 1.28 to 1.87 mg/g FW across treatments and years (**Figure 5C**). There were no significant interactions among treatments, and only the main effects of virus status (*p* = 0.032) and year (*p* = 0.016) returned significant results. There was a 17% reduction in seed tannins in GRBV+ vines compared to GRBV- vines, with no irrigation effect when averaged across years (**Table 7**).

**Table 7:** Main effects of irrigation treatment and virus status on seed phenolics variables averaged across years.

Treatment	Level	Seed tannins	Seed phenolics
		*——— mg/g berry FW ———*	
Irrigation	WW	1.55 ± 0.1	3.73 ± 0.3
	WD	1.51 ± 0.1	3.65 ± 0.3
	*p*-value	0.666	0.769
			
Virus	GRBV-	1.68 ± 0.1	4.35 ± 0.3
	GRBV+	1.40 ± 0.1	3.08 ± 0.3
	*p*-value	0.021	0.002

Data are means ± 1 standard error (n = 5). WW and WD are well-watered and water deficit irrigation treatments, respectively.

Seed total phenolic concentration ranged 1.5-fold from 3.03 to 4.40 mg/g FW across treatments and years (**Figure 5D**). Only the main effect of virus status returned significant results (*p* = 0.004). There was a 29% reduction in seed phenolics in GRBV+ vines compared to GRBV- vines, with no irrigation effect when averaged across years (**Table 7**).

## Discussion

The results of this study show that there is limited interaction between GRBV infection and water deficits, largely supporting the null hypothesis. In most variables tested, virus status and irrigation treatment independently affected vines, with a few notable exceptions. Virus effects were stronger but more seasonally variable, while water deficit effects were weaker but more seasonally consistent. Observed virus effects of reduced vine gas exchange and increased water status, coupled with reduced berry TSS and concentrations of phenolic compounds in skins and seeds confirm results from previous studies on different cultivars in different growing regions (Girardello et al., 2019; Martínez-Lüscher et al., 2019). In addition, the stronger virus effects and limited interaction with water deficits presented herein are similar to what has been shown in leafroll-infected grapevines (El Aou-ouad et al., 2016; El Aou-ouad et al., 2017).

### Temporal Distribution of GRBD Foliar Symptoms

GRBV infection consistently increased postveraison Ψ_stem_ regardless of the level of vine water status imposed by the irrigation treatments or season, and this occurred after decreases in postveraison leaf gas exchange. Decreases in leaf gas exchange themselves followed the appearance of the first foliar symptoms (leaf reddening). Moreover, the data show that significant virus effects on A_net_ became apparent prior to those on g_s_ independent of irrigation treatment. Martínez-Lüscher et al. (2019) similarly showed an increase in postveraison Ψ_stem_ in Cabernet Sauvignon vines across two rootstocks, and though they did not report any disease symptom severity data, they did report antecedent decreases in leaf gas exchange. While the data reported herein to some extent lack the temporal resolution required to firmly establish the order of events related to GRBD foliar symptom expression and progression, there appears to be a clearer picture emerging regarding the time course of GRBV-induced symptoms on vine physiology. Specifically, decreases in leaf gas exchange and appearance of red-leaf foliar symptoms precede the increases in vine water status, suggesting that it is the reduction in stomatal conductance that increases vine water status.

Decreases in leaf gas exchange in GRBV+ vines have been hypothesized to be a response to an accumulation of leaf carbohydrates that result in feedback inhibition of carbon assimilation (Martínez-Lüscher et al., 2019). This mechanism would be similar to that which has been recently shown in vines infected with *Grapevine leafroll-associated virus*, another phloem-limited virus with similar symptomology (Halldorson and Keller, 2018). In general, feedback inhibition of carbon assimilation and synthesis of anthocyanins in leaves in response to foliar sugar accumulation has been well-documented in grapevine (Downton et al., 1987; Roper and Williams, 1989; Quereix et al., 2001; Gutha et al., 2010). Halldorson and Keller (2018) also showed that accumulation of anthocyanins in leaves preceded other signs of senescence. Therefore, it is likely that GRBV effects on leaf carbohydrate metabolism and gas exchange precede the foliar accumulation of anthocyanins, suggesting that these flavonoids act as a photoprotectant of the photosynthetic apparatus that is unable to quench the excess light energy through normal photochemistry (Feild et al., 2001).

### Water Deficits Do Not Advance the Onset of Symptoms but May Advance Progression

Despite the differences in preveraison water status between WW and WD treatments, there were no differences in the timing of symptom onset within either year. Moreover, the severity of preveraison water deficits did not impact the onset of symptoms in the hypothesized direction –earlier symptoms resulting from more severe preveraison water deficits – but rather in the opposite direction. In 2017, average preveraison Ψ_stem_ ≈ -0.68 MPa across treatments, yet foliar symptoms were first observed approximately two weeks preveraison (25 July); while in 2018, average preveraison Ψ_stem_ ≈ -1.12 MPa across treatments, and symptoms were observed approximately at veraison (5 August). Since the appearance of foliar symptoms of GRBD are dependent on vine phenology (Blanco-Ulate et al., 2017), and preveraison water deficits have been shown to accelerate coloration of berries at veraison (Castellarin et al., 2007; Herrera and Castellarin, 2016), this result suggests that the onset of foliar symptoms is dependent on factors other than water status such as carbohydrate/nutrient status and/or solar radiation. Although vine nutrient status was not monitored in this study, it should be noted that 2018 was characterized by wildfire smoke that significantly reduced solar radiation during the preveraison period that could have delayed the onset of symptoms in that year.

While water deficits did not advance foliar symptom onset, they may advance symptom progression if they are severe. The absolute level of water status may in part account for why the rate of symptom progression was advanced in 2018 compared to 2017. In 2018, WD vines had a substantially lower water status on average (Ψ_stem_ ≈ -1.4 MPa) than in 2017 (Ψ_stem_ ≈ -0.9 MPa), and disease symptom severity progressed at a faster rate in those vines compared to WW vines. By comparison, WW vines in 2018 had a similar water status to WD vines in 2017. Thus, it is likely that disease symptom progression is only advanced under severe water deficits (Ψ_stem_ < -0.9 MPa), and explains why no differences were observed between irrigation treatments in 2017. However, the lack of resolution in the data precludes any further precision regarding Ψ_stem_ thresholds for GRBD severity progression.

### Short-Term Responses of Vine Growth

This study showed that overall vine vigor is not inhibited by GRBV infection in the short term. There was no response of any of the vegetative growth variables to virus status in either year, with only a small effect of irrigation treatment on pruning mass in 2018—likely due to the larger differences in vine water status that year. Recently, Martínez-Lüscher et al. (2019) showed no GRBD effect on vine pruning mass in Cabernet Sauvignon, while Reynard et al. (2018) reported pruning mass reductions of 33–35% in Gamay. Considering that leaf gas exchange was not reduced in GRBV+ vines preveraison in this study and in Martínez-Lüscher et al. (2019), and that most shoot elongation and canopy development occurs during this time (Matthews et al., 1987), it seems unlikely that GRBV infection would negatively impact vegetative growth unless there were other confounding biotic or abiotic factors.

Most reproductive growth variables in GRBV+ vines were similarly not affected over the 2-year study. Importantly, from a production perspective, yields were not reduced, and were in fact slightly increased in GRBV+ vines due to larger clusters made up of larger berries. Martínez-Lüscher et al. (2019) also reported some increases in berry size in response to GRBV infection in Cabernet Sauvignon for one of two rootstocks, though the differences were not apparent at commercial harvest. The larger berries in GRBV+ vines in this study were likely in part due to a higher postveraison water status that is well-known to increase berry size (Matthews and Anderson, 1989). However, it is unclear whether or not the differences in berry size due to GRBV-infection were established preveraison.

### Potential Long-Term Productivity Decline

The interaction between irrigation treatment and virus status on berries per cluster coupled with a lack of any treatment effects on clusters per shoot supports a hypothesis of late-season disruption of whole-vine carbohydrate metabolism by GRBV. Furthermore, this disruption seems to be exacerbated by water deficits. This suggests that long-term productivity of grapevines infected by GRBV may decline if subjected to repeated water deficits postveraison. However, it should be noted that there were no effects of virus (nor any interaction with water deficits) on yield in this study, despite the fact that data vines were likely infected with GRBV prior to its initiation.

The number of clusters per shoot is established preveraison and is sensitive to water deficits during this period, with few differences among cultivars (Williams, 2000; Levin et al., 2020). The lack of clusters per shoot response in this study parallels the lack of preveraison virus effects or severe water deficits. In contrast, the number of berries per cluster is established postveraison and into the following growing season, and depends more strongly on cultivar (Keller and Tarara, 2010; Levin et al., 2020). The increase in berries per cluster of WD/GRBV- vines in this study can be attributed to a shift in carbohydrate partitioning to reproductive organs during water deficits (Yakushiji et al., 1998), and that water deficits in this study were more pronounced postveraison. Concomitantly, berries per cluster of WD/GRBV+ vines were either not different or reduced compared to WW/GRBV+ vines, providing evidence for a disruption of this mechanism. Thus, GRBV infection may potentially negatively impact carbohydrate allocation to sinks other than the fruit, and the worsening of this inhibition under water deficit suggests potential future declines in vine productivity that were not observed in this study.

The lack of yield response to GRBV found in this study has been previously reported in studies characterizing GRBV effects on vine performance (Reynard et al., 2018; Martínez-Lüscher et al., 2019). Those studies, as in this one, were also conducted on mature (> 5 years old) vines that were likely infected prior to their use in the research; however, it is unclear how many years before. The reductions in net carbon assimilation (and disruption of translocation) due to GRBV infection may be at least partially offset by stored carbohydrate reserves in woody tissue. Accordingly, these reserves would be mobilized to support continuted reproductive development and yield maintenance in (recently) infected vines. It is certainly possible, therefore, that GRBV infection may not cause long-term productivity decline if vines were infected once mature, but it remains unclear to what degree management practices post-infection and/or vine age at time of infection may play a role.

### Consistent Response of TSS, Inconsistent Acids

The consistent response of TSS to GRBV infection across years and irrigation treatments, coupled with the lack of response of berry pH and TA across all treatments underscores that GRBV infection has a direct role in the inhibition of berry sugar accumulation, compared to a likely indirect role in berry organic acid metabolism. Effects of GRBV infection on primary berry chemistry have repeatedly been reported as reduced TSS and pH together with increased TA (Sudarshana et al., 2013; Sudarshana et al., 2015; Sudarshana and Zalom, 2017). However, in this study, TSS was consistently reduced in two consecutive years due to viral infection, while pH and TA were relatively unaffected.

Recent work across varied cultivars and regions has also shown that reduced TSS is a more consistent and stronger symptom of GRBV infection compared to that of increased TA or reduced pH (Reynard et al., 2018; Girardello et al., 2019; Martínez-Lüscher et al., 2019). For example, Reynard et al. (2018) reported significantly *lower* TA in GRBV+ Gamay vines over three consecutive seasons in Switzerland. Girardello et al. (2019) reported primary berry chemistry data for nine pairs of GRBV+/- samples across three cultivars and two years in California, and though the authors did not conduct statistical analyses nor provide any measures of dispersion in the data, more than half of the pairs had equivalent pH values and TA values of only +0.5 g/L in their GRBV+ samples—well within the confidence interval of the TA contrast estimate between GRBV+/- fruit in this study. Finally, Martínez-Lüscher et al. (2019) showed that berry pH was similar between GRBV+/- Cabernet Sauvignon samples at harvest. Yet, in all of the aforementioned studies, as reported herein, TSS was consistently and significantly reduced in GRBV+ in nearly all cases.

The lack of interaction between irrigation treatment and virus status on TSS—and relatively small effect of irrigation treatment—in both years shows that GRBV infection has a strong influence on the ripening program that cannot be overridden by applying water deficits. Moderate water deficits typically advance ripening in grape berries compared to WW controls (Castellarin et al., 2007), likely due to a drought-induced change in carbohydrate partitioning (Yakushiji et al., 1998), thus it was surprising that the applied water deficits in this study somewhat reduced TSS at harvest. Nevertheless, the effect was conserved across both GRBV+ and GRBV- vines in this study, providing some evidence that water deficits have no beneficial, if not an altogether deleterious, effect on ripening in GRBV+ fruit.

### Virus Effects on Phenolics in Berry Skins Highly Dependent on Environment

When considering the responses of secondary metabolites in berry skins, a common result was that the effect of GRBV infection on anthocyanins and tannins depended strongly on year. In general, high year-to-year and site-to-site variability regarding the effects of GRBV infection on berry secondary metabolites has been previously reported (Girardello et al., 2019; Martínez-Lüscher et al., 2019). While the data shown in this study corroborate these findings, they do not resolve the question of why the effect of GRBV infection on secondary metabolites varies so much year-to-year.

The negative effect of GRBV on anthocyanin concentration was not as strong in 2018 compared to 2017, and tannins were actually increased in GRBV+ berries in 2018 compared to 2017, when averaged across irrigation treatments. Indeed, the change in pruning practice from 2017 to 2018 may have altered aspects of the cluster microclimate such as light environment, which is known to be highly influential on development berry secondary metabolites, particularly in Pinot noir (Dokoozlian and Kliewer, 1996; Feng et al., 2015). However, the ANCOVA model including shoots per vine was fit in an attempt to account in part for these differences, and did not result in a significantly better fit compared to the simpler model without this co-variable. Thus, if cluster microclimate was altered by pruning practices such that it influenced berry secondary metabolism in a significant way, then the shoots per vine variable did not sufficiently capture this effect.

### Water Deficits Inhibit Secondary Metabolism In GRBV+ Berry Skins

In GRBV+ vines, water deficits had similar effects on skin tannins across years, but their effects on anthocyanins depended (somewhat) on year. This was likely related to the temporal separation of (1) the development of these two compound classes within the berry skin, and (2) the severity of the water deficits themselves. Although both classes of phenolic compounds are products of the phenylpropanoid pathway, tannins are largely synthesized preveraison, whereas anthocyanins are formed postveraison (Adams, 2006). Regardless of their developmental separation in time however, biosynthesis of both compounds in berry skins has been shown to be advanced under water deficits—in healthy vines (Castellarin et al., 2007). This phenomenon was also demonstrated in this study, whereby both tannin and anthocyanin concentrations were increased in healthy vines experiencing a water deficit. However, in GRBV+ vines, water deficits reduced tannin concentration in berry skins in both years, and only marginally increased anthocyanin concentration in 2018. In that year, water deficits and disease severity were greater and lower compared to 2017, respectively, at least partially explaining the year-to-year difference in anthocyanin concentration. Therefore, though the disruption of normal secondary metabolism during berry ripening in GRBV+ vines (Blanco-Ulate et al., 2017) can be somewhat overcome by applying a water deficit, it depends on the severity of said deficit, and will ultimately not be entirely ameliorated. Finally, that the concentration of tannins in berry skins is reduced by water deficits in GRBV+ vines suggests that the virus is negatively impacting berry development preveraison – before any disease symptoms are visible.

### Secondary Metabolism in Berry Seeds Controlled Solely by GRBV

Tannins and total phenolic concentrations in berry seeds were strongly reduced by GRBV infection, with no effect of water deficits and minimal year effects. Since grape seed formation and development are largely completed preveraison (Ristic and Iland, 2005), the data reported in this study further support the aforementioned suggestion that GRBV negatively affects berry development during this time. Furthermore, differences in preveraison vine water status between irrigation treatments within years, and across treatments between years did not manifest in any differences in general seed phenolic concentrations, thus solidifying the strong control of GRBV infection on seed development. The large amount of carbon required to synthesize phenolic compounds in lignified structures such as berry seeds (Adams, 2006) and the ultimate inhibition of said synthesis support the hypothesis that the link between vine carbon metabolism and phenolic biosynthesis is broken by GRBV infection (Martínez-Lüscher et al., 2019). Finally, considering that the proportion of whole berry phenolics of each class from seeds is larger than those from skins, the consequences of significant reductions in seed phenolics in GRBV+ berries may override any biotic or abiotic effects on skins from an enological and wine sensory perspective.

## Conclusion

This study was the first to test the hypothesis that there is an interactive effect of water deficits and GRBV infection on GRBD severity, vine growth, and fruit quality in field-grown grapevines. In general, water deficits and virus status did not strongly interact on most tested variables, with a few exceptions. Each treatment factor acted more or less independently, with virus effects predominating over water deficit effects. Furthermore, while water deficits tended to impact tested variables consistently over two years, virus effects varied more strongly with year.

From a practical perspective, the results of this study show that applying water deficits to GRBV-infected grapevines will not improve fruit quality in Pinot noir grapevines. In some cases, water deficits may worsen fruit quality, but this may depend on year and severity of the water deficit itself. From a biological perspective, this study confirmed that the negative effects of GRBD are strongly controlled by GRBV, likely at the molecular level, and shows that this control is unable to be reversed by any water deficit. The virus changes vine carbon metabolism from the leaf (assimilation) to the whole-vine (partitioning) level that in turn negatively impacts secondary metabolism in the short term and potentially vine productivity in the long-term.

## Data Availability Statement

The raw data supporting the conclusions of this article will be made available by the authors, without undue reservation.

## Author Contributions

AL and AK designed the study, collected and analyzed the data, and wrote the manuscript.

## Funding

This work was supported in part by the American Vineyard Foundation (AVF 2017-2097 and 2018-2097), the Oregon Department of Agriculture Specialty Crop Block Grant Program (ODA 2017-5011-K11720), and the Rogue Valley Winegrowers Association.

## Conflict of Interest

The authors declare that the research was conducted in the absence of any commercial or financial relationships that could be construed as a potential conflict of interest.

## References

[B1] Adams D. O. (2006). Phenolics and Ripening in Grape Berries. Am. J. Enol. Viticult. 57 (3), 249–256.

[B2] Al Rwahnih M. Dave A. Anderson M. M. Rowhani A. Uyemoto J. K. Sudarshana M. R. (2013). Association of a DNA Virus with Grapevines Affected by Red Blotch Disease in California. Phytopathology 103 (10), 1069–1076. 10.1094/Phyto-10-12-0253-R 23656312

[B3] Al Rwahnih M. Rowhani A. Golino D. A. Islas C. M. Preece J. E. Sudarshana M. R. (2015). Detection and genetic diversity of Grapevine red blotch-associated virus isolates in table grape accessions in the National Clonal Germplasm Repository in California. Can. J. Plant Pathol. 37 (1), 130–135. 10.1080/07060661.2014.999705

[B4] Bahder B. W. Zalom F. G. Jayanth M. Sudarshana M. R. (2016). Phylogeny of Geminivirus Coat Protein Sequences and Digital PCR Aid in Identifying Spissistilus festinus as a Vector of Grapevine red blotch-associated virus. Phytopathology 106 (10), 1223–1230. 10.1094/phyto-03-16-0125-fi 27111804

[B5] Blanco-Ulate B. Hopfer H. Figueroa-Balderas R. Ye Z. R. Rivero R. M. Albacete A. (2017). Red blotch disease alters grape berry development and metabolism by interfering with the transcriptional and hormonal regulation of ripening. J. Exp. Bot. 68 (5), 1225–1238. 10.1093/jxb/erw506 28338755 PMC5444480

[B6] Bortolami G. Gambetta G. A. Delzon S. Lamarque L. J. Pouzoulet J. Badel E. (2019). Exploring the Hydraulic Failure Hypothesis of Esca Leaf Symptom Formation. Plant Physiol. 181 (3), 1163–1174. 10.1104/pp.19.00591 31455632 PMC6836855

[B7] Buchs N. Braga-Lagache S. Uldry A. C. Brodard J. Debonneville C. Reynard J. S. (2018). Absolute Quantification of Grapevine Red Blotch Virus in Grapevine Leaf and Petiole Tissues by Proteomics. Front. Plant Sci. 9:1735. 10.3389/fpls.2018.01735 30555495 PMC6281998

[B8] Calvi B. L. (2011). Effects Of Red-leaf Disease On Cabernet Sauvignon At The Oakville Experimental Vineyard And Mitigation By Harvest Delay And Crop Adjustment (University of California - Davis: M.S. Master’s Thesis).

[B9] Casassa L. F. Keller M. Harbertson J. F. (2015). Regulated Deficit Irrigation Alters Anthocyanins, Tannins and Sensory Properties of Cabernet Sauvignon Grapes and Wines. Molecules 20 (5), 7820–7844. 10.3390/molecules20057820 25939070 PMC6272144

[B10] Castellarin S. D. Matthews M. A. Di Gaspero G. Gambetta G. A. (2007). Water deficits accelerate ripening and induce changes in gene expression regulating flavonoid biosynthesis in grape berries. Planta 227 (1), 101–112. 10.1007/s00425-007-0598-8 17694320

[B11] Choat B. Gambetta G. A. Wada H. Shackel K. A. Matthews M. A. (2009). The effects of Pierce’s disease on leaf and petiole hydraulic conductance in Vitis vinifera cv. Chardonnay. Physiol. Plant 136 (4), 384–394. 10.1111/j.1399-3054.2009.01231.x 19470095

[B12] Choi H. K. Iandolino A. da Silva F. G. Cook D. R. (2013). Water Deficit Modulates the Response of Vitis vinifera to the Pierce’s Disease Pathogen Xylella fastidiosa. Mol. Plant-Microbe Interact. 26 (6), 643–657. 10.1094/mpmi-09-12-0217-r 23425100

[B13] Cieniewicz E. J. Pethybridge S. J. Gorny A. Madden L. V. McLane H. Perry K. L. (2017). Spatiotemporal spread of grapevine red blotch-associated virus in a California vineyard. Virus Res. 241, 156–162. 10.1016/j.virusres.2017.03.020 28392444

[B14] Cieniewicz E. Perry K. L. L. Kruse A. Cilia M. Fuchs M. (2018). Insights into the epidemiology and transmission of grapevine red blotch virus. Phytopathology 108 (10), 94–102. 10.1094/PHYTO-07-17-0239-R 28945519

[B15] Dalton D. T. Hilton R. J. Kaiser C. Daane K. M. Sudarshana M. R. Vo J. (2019). Spatial Associations of Vines Infected With Grapevine Red Blotch Virus in Oregon Vineyards. Plant Dis. 103 (7), 1507–1514. 10.1094/pdis-08-18-1306-re 31025904

[B16] Dokoozlian N. K. Kliewer W. M. (1996). Influence of Light on Grape Berry Growth and Composition Varies during Fruit Development. J. Am. Soc. Hortic. Sci. 121 (5), 869–874. 10.21273/JASHS.121.5.869

[B17] Downton W. J. S. Grant W. J. R. Loveys B. R. (1987). Diurnal changes in the photosynthesis of field-grown grape vines. New Phytol. 105, 71–80. 10.1111/j.1469-8137.1987.tb00111 33874032

[B18] El Aou-ouad H. Montero R. Medrano H. Bota J. (2016). Interactive effects of grapevine leafroll-associated virus 3 (GLRaV-3) and water stress on the physiology of Vitis vinifera L. cv. Malvasia de Banyalbufar and Giro-Ros. J. Plant Physiol., 196–197, 106-115. 10.1016/j.jplph.2016.04.003 27153513

[B19] El Aou-ouad H. Pou A. Tomas M. Montero R. Ribas-Carbo M. Medrano H. (2017). Combined effect of virus infection and water stress on water flow and water economy in grapevines. Physiol. Plantarum 160 (2), 171–184. 10.1111/ppl.12541 28044321

[B20] Feild T. S. Lee D. W. Holbrook N. M. (2001). Why Leaves Turn Red in Autumn. The Role of Anthocyanins in Senescing Leaves of Red-Osier Dogwood. Plant Physiol. 127 (2), 566–574. 10.1104/pp.010063 11598230 PMC125091

[B21] Feng H. Yuan F. Skinkis P. A. Qian M. C. (2015). Influence of cluster zone leaf removal on Pinot noir grape chemical and volatile composition. Food Chem. 173, 414–423. 10.1016/j.foodchem.2014.09.149 25466040

[B22] Gambetta G. A. Fei J. Rost T. L. Matthews M. A. (2007). Leaf scorch symptoms are not correlated with bacterial populations during Pierce’s disease. J. Exp. Bot. 58 (15-16), 4037–4046. 10.1093/jxb/erm260 18037677

[B23] Girardello R. C. Cooper M. L. Smith R. J. Lerno L. A. Bruce R. C. Eridon S. (2019). Impact of Grapevine Red Blotch Disease on Grape Composition of Vitis vinifera Cabernet Sauvignon, Merlot, and Chardonnay. J. Agric. Food Chem. 67 (19), 5496–5511. 10.1021/acs.jafc.9b01125 31013081

[B24] Greenspan M. D. Shackel K. A. Matthews M. A. (1994). Developmental-Changes in the Diurnal Water-Budget of the Grape Berry Exposed to Water Deficits. Plant Cell Environ. 17 (7), 811–820. 10.1111/j.1365-3040.1994.tb00175.x

[B25] Gutha L. R. Cassasa L. F. Harbertson J. F. Naidu R. (2010). Modulation of flavonoid biosynthetic pathway genes and anthocyanins due to virus infection in grapevine (*Vitis vinifera* L.) leaves. BMC Plant Biol. 10, 187. 10.1186/1471-2229-10-187 20731850 PMC2956537

[B26] Halldorson M. M. Keller M. (2018). Grapevine leafroll disease alters leaf physiology but has little effect on plant cold hardiness. Planta 248 (5), 1201–1211. 10.1007/s00425-018-2967-x 30094489

[B27] Harbertson J. F. Mireles M. Yu Y. (2015). Improvement of BSA Tannin Precipitation Assay by Reformulation of Resuspension Buffer. Am. J. Enol. Viticult. 66 (1), 95–99. 10.5344/ajev.2014.14082

[B28] Heredia T. M. Adams D. O. Fields K. C. Held P. G. Harbertson J. F. (2006). Evaluation of a Comprehensive Red Wine Phenolics Assay Using a Microplate Reader. Am. J. Enol. Viticult. 57 (4), 497–502.

[B29] Herrera J. C. Castellarin S. D. (2016). Preveraison Water Deficit Accelerates Berry Color Change in Merlot Grapevines. Am. J. Enol. Viticult. 67 (3), 356–360. 10.5344/ajev.2016.15083

[B30] Horsfall J. G. Barratt R. W. (1945). An improved grading system for measuring plant diseases (Abstract). Phytopathoglogy 35, 655. 10.1094/Phyto-35-655 40934413

[B31] Keller M. Tarara J. M. (2010). Warm spring temperatures induce persistent season-long changes in shoot development in grapevines. Ann. Bot. 106 (1), 131–141. 10.1093/aob/mcq091 20513742 PMC2889799

[B32] Krenz B. Thompson J. R. Fuchs M. Perry K. L. (2012). Complete Genome Sequence of a New Circular DNA Virus from Grapevine. J. Virol. 86 (14), 7715–7715. 10.1128/jvi.00943-12 22733880 PMC3416304

[B33] Krenz B. Thompson J. R. McLane H. L. Fuchs M. Perry K. L. (2014). Grapevine red blotch-associated virus Is Widespread in the United States. Phytopathology 104 (11), 1232–1240. 10.1094/phyto-02-14-0053-r 24805072

[B34] Kuznetsova A. Brockoff P. B. Rune H. B. (2017). lmerTest Package: Tests in Linear Mixed Effects Models. J. Stat. Softw. 82 (13), 1–26. 10.18637/jss.v082.i13

[B35] Lenth R. (2019). emmeans: Estimated Marginal Means, aka Least-Squares Means. R package version 1.4.3. Available at: https://CRAN.R-project.org/package=emmeans.

[B36] Levin A. D. Matthews M. A. Williams L. E. (2020). Effect of Preveraison Water Deficits on the Yield Components of 15 Winegrape Cultivars. Am. J. Enol. Viticult. 71 (3), 208–221. 10.5344/ajev.2020.19073

[B37] Levin A. D. (2019). Re-evaluating pressure chamber methods of water status determination in field-grown grapevine (Vitis spp.). Agric. Water Manage. 221, 422–429. 10.1016/j.agwat.2019.03.026

[B38] Martínez-Lüscher J. Plank C. M. Brillante L. Cooper M. L. Smith R. J. Al-Rwahnih M. (2019). Grapevine Red Blotch Virus May Reduce Carbon Translocation Leading to Impaired Grape Berry Ripening. J. Agric. Food Chem. 67 (9), 2437–2448. 10.1021/acs.jafc.8b05555 30721055

[B39] Matthews M. A. Anderson M. M. (1988). Fruit Ripening in Vitis vinifera L.: Responses to Seasonal Water Deficits. Am. J. Enol. Viticult. 39 (4), 313–320.

[B40] Matthews M. A. Anderson M. M. (1989). Reproductive Development in Grape (*Vitis vinifera* L.): Responses to Seasonal Water Deficits. Am. J. Enol. Viticult. 40 (1), 52–60.

[B41] Matthews M. A. Anderson M. M. Schultz H. R. (1987). Phenologic and growth responses to early and late season water deficits in Cabernet franc. Vitis 26, 147–160.

[B42] Preto C. R. Sudarshana M. R. Bollinger M. L. Zalom F. G. (2018a). Vitis vinifera (Vitales:Vitaceae) as a Reproductive Host of Spissistilus festinus (Hemiptera: Membracidae). J. Insect Sci. 18 (6), 1–7. 10.1093/jisesa/iey129 PMC6299463 30566644

[B43] Preto C. R. Sudarshana M. R. Zalom F. G. (2018b). Feeding and Reproductive Hosts of Spissistilus festinus (Say) (Hemiptera: Membracidae) Found in Californian Vineyards. J. Econom. Entomol. 111 (6), 2531–2535. 10.1093/jee/toy236 30107410

[B44] Quereix A. Dewar R. C. Gaudillere J. P. Dayau S. Valancogne C. (2001). Sink feedback regulation of photosynthesis in vines: measurements and a model. J. Exp. Bot. 52 (365), 2313–2322. 10.1093/jexbot/52.365.2313 11709581

[B45] Reynard J. S. Brodard J. Dubuis N. Zufferey V. Schumpp O. Schaerer S. (2018). Grapevine red blotch virus: Absence in Swiss Vineyards and Analysis of Potential Detrimental Effect on Viticultural Performance. Plant Dis. 102 (3), 651–655. 10.1094/pdis-07-17-1069-re 30673492

[B46] Ricketts K. D. Gomez M. I. Fuchs M. F. Martinson T. E. Smith R. J. Cooper M. L. (2017). Mitigating the Economic Impact of Grapevine Red Blotch: Optimizing Disease Management Strategies in US Vineyards. Am. J. Enol. Viticult. 68 (1), 127–135. 10.5344/ajev.2016.16009

[B47] Ristic R. Iland P. G. (2005). Relationships between seed and berry development of *Vitis Vinifera* L. cv Shiraz: Developmental changes in seed morphology and phenolic composition. Aust. J. Grape Wine Res. 11, 43–58. 10.1111/j.1755-0238.2005.tb00278.x

[B48] Romero J. L. Carver G. D. Johnson P. A. Perry K. L. Thompson J. R. (2019). A rapid, sensitive and inexpensive method for detection of grapevine red blotch virus without tissue extraction using loop-mediated isothermal amplification. Arch. Virol. 164 (5), 1453–1457. 10.1007/s00705-019-04207-y 30895404

[B49] Roper T. R. Williams L. E. (1989). Net CO2 assimilation and carbohydrate partitioning of grapevine leaves in response to trunk girdling and gibberellic acid application. Plant Physiol. 89, 1136–1140. 10.1104/pp.89.4.1136 16666676 PMC1055987

[B50] Sudarshana M. Zalom F. G. (2017). Grapevine red blotch disease, an emerging problem for wine grape production in the US. Phytopathology 107 (12), 193–193. 10.1094/PHYTO-12-14-0369-FI

[B51] Sudarshana M. R. Gonzalez A. Dave A. Wei A. Smith R. Anderson M. M. (2013). Grapevine red blotch-associated virus is widespread in California and US vineyards. Phytopathology 103 (6), 140–140. 10.1094/PHYTO-103-6-S2.1

[B52] Sudarshana M. R. Perry K. L. Fuchs M. F. (2015). Grapevine Red Blotch-Associated Virus, an Emerging Threat to the Grapevine Industry. Phytopathology 105 (7), 1026–1032. 10.1094/phyto-12-14-0369-fi 25738551

[B53] Wallis C. M. Sudarshana M. R. (2016). Effects of Grapevine red blotch-associated virus (GRBaV) infection on foliar metabolism of grapevines. Can. J. Plant Pathol. 38 (3), 358–366. 10.1080/07060661.2016.1227374

[B54] Wickham H. (2016). “ggplot2: Elegant Graphics for Data Analysis”. (New York: Springer-Verlag).

[B55] Williams L. E. Matthews M. A. (1990). ““Grapevine,”,” in Irrigation of Agricultural Crops. Eds. Steward B. A. Nielsen D. R. (Madison, WI: American Society of Agronomy), 1019–1055.

[B56] Williams L. E. Grimes D. W. Phene C. J. (2010a). The effects of applied water at various fractions of measured evapotranspiration on reproductive growth and water productivity of Thompson Seedless grapevines. Irrigation Sci. 28 (3), 233–243. 10.1007/s00271-009-0173-0

[B57] Williams L. E. Grimes D. W. Phene C. J. (2010b). The effects of applied water at various fractions of measured evapotranspiration on water relations and vegetative growth of Thompson Seedless grapevines. Irrigation Sci. 28 (3), 221–232. 10.1007/s00271-009-0171-2

[B58] Williams L. E. (2000). ““Bud Development and Fruitfulness of Grapevines,”,” in Raisin Production Manual. Ed. Christensen L. P. (Berkeley, CA: University of California), 24–29.

[B59] Williams L. E. (2012). Effects of applied water amounts at various fractions of evapotranspiration (ETc) on leaf gas exchange of Thompson Seedless grapevines. Aust. J. Grape Wine Res. 18 (1), 100–108. 10.1111/j.1755-0238.2011.00176.x

[B60] Williams L. E. (2014). Determination of Evapotranspiration and Crop Coefficients for a Chardonnay Vineyard Located in a Cool Climate. Am. J. Enol. Viticult. 65 (2), 159–168. 10.5344/ajev.2014.12104

[B61] Yakushiji H. Morinaga K. Nonami H. (1998). Sugar Accumulation and Partitioning in Satsuma Mandarin Tree Tissues and Fruit in Response to Drought Stress. J. Am. Soc. Hortic. Sci. 123 (4), 719–726. 10.21273/JASHS.123.4.719

[B62] Yepes L. M. Cieniewicz E. Krenz B. McLane H. Thompson J. R. Perry K. L. (2018). Causative Role of Grapevine Red Blotch Virus in Red Blotch Disease. Phytopathology 108 (7), 902–909. 10.1094/phyto-12-17-0419-r 29436986

